# Special Issue: DNA Helicases: Mechanisms, Biological Pathways, and Disease Relevance

**DOI:** 10.3390/genes12030356

**Published:** 2021-03-01

**Authors:** Robert M. Brosh

**Affiliations:** Section on DNA Helicases, Translational Gerontology Branch, National Institute on Aging, NIH, Baltimore, MD 21224, USA; broshr@mail.nih.gov

**Keywords:** helicase, replication, DNA repair, recombination, transcription, DNA structure, aging, cancer, genetic disease, genomic stability

DNA helicases have emerged as a prominent class of nucleic acid-metabolizing enzymes that play important roles in genome maintenance and cellular homeostasis. DNA helicases not only play essential roles in replication, DNA repair, and recombination, but they also influence gene expression and chromosome structure and act to resolve dynamic DNA structures that pose a source of genomic instability. Although the basic mechanisms of the unwinding of structured nucleic acids by DNA helicases share some features of commonality, research shows that there are specialized DNA-unwinding mechanisms that can be regulated by assembly states, post-translational modifications, and protein interactions.

With the discovery of genetic disorders linked to helicase gene mutations and various cancers associated with helicase defects, it has become more important than ever to describe and characterize the molecular and cellular functions and pathways of DNA helicases to gain new understanding and move toward translational approaches in medicine.

A Special Issue of *Genes* entitled DNA Helicases: Mechanisms, Biological Pathways, and Disease Relevance is dedicated to a compilation of articles on DNA helicases from leading experts in the field. This *Genes* Special Issue provides diverse and informative perspectives on a spectrum of DNA helicase proteins and teaches lessons about their molecular and genetic functions and, more broadly, their roles in genome homeostasis.

*History of DNA Helicases*, an article by Brosh and Matson, sets the table for the *Genes* Special Issue by providing an in-depth and comprehensive assessment of how the study of helicases has progressed since their first discovery nearly 45 years ago [1]. Innovative analyses of viral, prokaryotic, and eukaryotic helicases from many labs over the years are discussed in terms of their biochemical/biophysical, structural, and molecular properties as well as genetic studies of how these DNA-unwinding enzymes function in vivo. By telling the story of how the field has developed and continues to push forward in the 21st century, we aimed to describe new insights into the roles of DNA helicases in genome biology and their emergence as a key class of proteins in human diseases, aging, and cancer.

So much of our knowledge of how helicases operate has been gleaned from seminal biochemical and genetic studies with bacterial proteins. Bianco addresses, in his review article, an in-depth discussion of the complex interactive and helicase-centric processes that occur at stalled replication forks in *E. coli* [2]. Depending on the type of lesion (a single-stranded break, double-stranded break, DNA crosslink, or static protein–DNA complex), the bacterial cell elicits different responses and mechanisms to deal with the potentially mutagenic and, in some cases, lethal damage. Bianco’s expertise and insightful commentary on the complexity of the interactions of DNA helicases with single-stranded DNA-binding proteins to deal with stalled replication forks in this context are a highly valuable addition to the *Genes* Special Issue.

One of the more prominent families of DNA helicases in terms of disease relevance is the RecQ grouping. Gupta and Schmidt provide an excellent launchpad for considering the important roles of RecQ helicases by providing a comprehensive assessment of the RecQ helicases in the model genetic organisms *Saccharomyces cerevisiae* and *Saccharomyces pombe* [3]. With such a strong degree of conservation in many of the roles that these RecQ helicases play in the unicellular yeast organism, the review from Schmidt’s lab helps readers to appreciate and understand the complex genetic pathways and cellular roles of these RecQ helicases, which serve as a highly useful model for the study of higher eukaryotic RecQ orthologs.

Although there have been extensive studies of the three human RECQ helicases encoded by genes in which biallelic mutations are linked to diseases characterized by chromosomal instability, premature aging, and cancer, there is much less known about the two remaining human RECQ helicases: RECQ(L)1 and RECQ(L)5. Therefore, I was highly pleased when Drs. Sharma [4] and Janscak [5], two leaders in the analyses of these respective helicases, both agreed to contribute. I think that readers will find both review articles very informative because they delve into the latest understanding of each helicase’s unique importance. Sharma’s article on RECQ(L)1 helps to establish its significant role in genome maintenance and the suppression of breast cancer [4]. Janscak’s article on RECQ(L)5 dissects the new understanding of its mechanism of action to help cells cope with replication–transcription conflicts [5]. The papers on RECQ(L)1 and RECQ(L)5 provide a new and creative appreciation of the genetic pathways in which these helicases operate, which until very recently, remained elusive.

Coming from a more global perspective, Huselid and Bunting take on the difficult but rewarding task of discussing, in their review article, how DNA helicases play diverse and complex roles in the regulation of homologous recombination, an important means for cells to use to heal lethal double-stranded breaks that arise from genotoxic insults or broken replication forks in a manner that suppresses their deleterious effects on chromosomal stability [6]. Both pro-recombinogenic and anti-recombinogenic roles of DNA helicases are discussed in a way that is intelligible and useful for both experts and those newer to the field of DNA repair and the replication stress response.

In addition to DNA repair pathways, certain specialized DNA helicases are important for their ability to resolve unusually structured nucleic acids that deviate from the conventional DNA double helix and potentially interfere with normal cellular DNA replication or transcription. Lansdorp and van Wietmarschen provide an insightful review article on how the Fe–S cluster helicases FANCJ (mutated in Fanconi anemia) and RTEL1 (mutated in Dyskeratosis congenita), as well as the RECQ helicase BLM (mutated in Bloom’s syndrome), act upon a unique four-stranded DNA structure known as a G-quadruplex (G4) to regulate gene expression and maintain genomic stability [7].

My own lab has taken a particular interest in the functions of FANCJ in nucleic acid metabolism and found that the helicase plays a vital role in smoothing out G4 DNA structures to allow efficient replication in human cells [8,9,10]. Consequently, I was highly enthusiastic for Wu’s lab to contribute a research article to the *Genes* Special Issue on DNA helicases describing FANCJ’s novel interaction with a cellular translesion polymerase and associated factor (PCNA) to synthesize past G4 obstacles [11]. With the emerging importance of G4 in genomic stability, gene regulation, telomere metabolism, and human disease, such experimental work advances our understanding of not only how G4 DNA is tolerated in vivo but also the complexity of G4-induced mutagenesis.

Another family of DNA helicases with profound roles in genome maintenance is represented by the PIF1 helicases. Muellner and Schmidt provide a critical analysis and summary of the PIF1 family of DNA helicases in yeast, providing an excellent foray into understanding their complex genetic and molecular roles [12]. By their narrative discussion of the involvement of PIF1 helicases in DNA repair and the replication stress response, the reader is informed in such a manner to appreciate their importance in the molecular-genetic pathways of higher eukaryotes.

In this *Genes* Special Issue on DNA helicases, the Bochman lab contributed a research article describing their findings from a molecular-genetic analysis of the *S. cerevisiae* Pif1 [13]. Although there is considerable effort to dissect the functional importance of its helicase activity, the researchers investigated how a noncatalytic domain of Pif1 modulates its function biochemically and in vivo. This work demonstrated that the loss of the N-terminal region diminished helicase activity and modulated the toxicity of overexpressed Pif1 in vivo. Evidence was also presented that the Pif1 N-terminal domain is important for the regulation of telomerase activity. Structure–function studies such as the Bochman lab’s using a model genetic organism continue to help to inform researchers and translate the findings to design experimentation for the investigation of mechanism(s) of action in human cells.

A conserved eukaryotic helicase of considerable interest, but less well studied than PIF1 family members, is DNA helicase B (HELB), the topic of a review article by the Byrd lab [14]. HELB is implicated in both the initiation of DNA replication and the DNA damage and replication stress response. Hazeslip et al. provide a comprehensive and current assessment of HELB’s domain structure, subcellular localization, functions and pathways, regulation, and gene variants. The information presented is a valuable resource to build upon for future studies and research hypotheses.

With the beneficial articles on eukaryotic nuclear DNA helicases, I was pleased to have the Falkenberg lab contribute a review on the mammalian replicative mitochondrial DNA helicase TWINKLE and other human mitochondrial DNA helicases [15]. Given the importance of TWINKLE mutations for human diseases characterized by mitochondrial defects, neurodegeneration, and accelerated aging, the review by Peter and Falkenberg is a highly significant contribution to the field. Moreover, the inclusion of a discussion of other mitochondrial helicases (PIF1, DNA2, RECQL4, and SUV3) is welcome and really provides a one-stop resource for those interested in the growing field of helicases involved in mitochondrial genome metabolism.

As communicated in our review, *History of DNA Helicases* [1], the field has gained its momentum from studies of a broad spectrum of helicase proteins encoded by genomes from different kingdoms as well as viruses. While the study of mammalian DNA helicases has grown over the last several decades because of the expanding interest in their roles in human disease, research in plant DNA helicases is beginning to blossom with advances in plant breeding and plant genome engineering to improve agriculture. Dorn and Puchta provide a unique and comprehensive review of plant DNA helicases that is a welcome resource in this rapidly developing area [16].

Overall, this collection of articles on DNA helicases provided in the *Genes* Special Issue represents a very comprehensive and significant source of information with testable models and emerging hypotheses to advance the field and bring a new cadre of scientists into the fold of helicase and nucleic acid metabolism research.
